# Supplementary figures and images for: Validation of low-density lipoprotein cholesterol equations in pediatric population
Source: PeerJ. 2023 Jan 5;11:e14544. doi: 10.7717/peerj.14544 (PMC9826611; doi:10.7717/peerj.14544)

Concordances of the different equations for LDL-C estimation by triglycerides strata

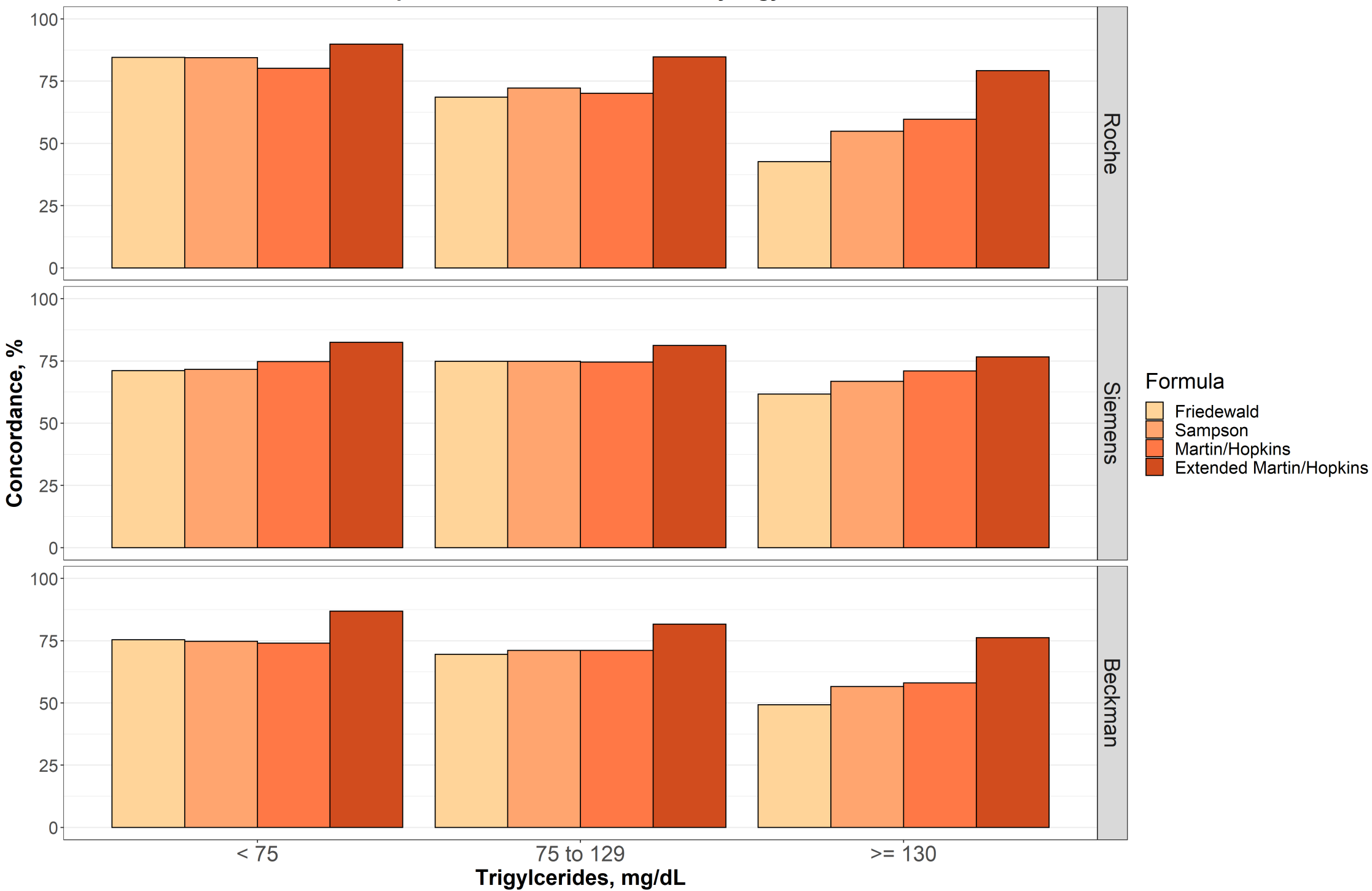

Supplement: Supplemental Information 1 [file peerj-11-14544-s001.pdf]

Concordances of the different equations for LDL-C estimation by nonHDL-C strata

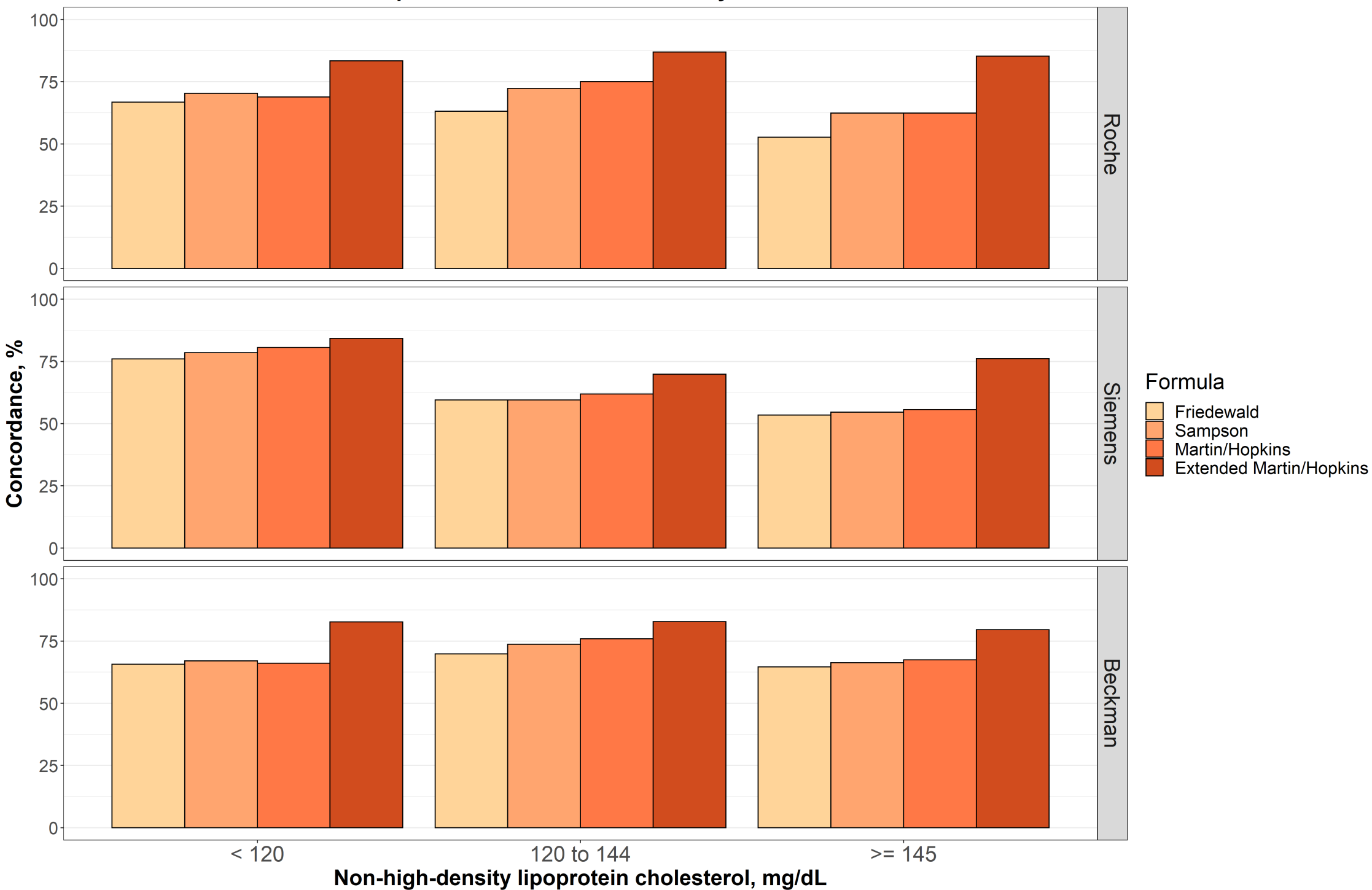

Supplement: Supplemental Information 5 [file peerj-11-14544-s005.pdf]

Concordances of the different equations for LDL-C estimation by nonHDL-C strata (LDL < 110 mg/dL)

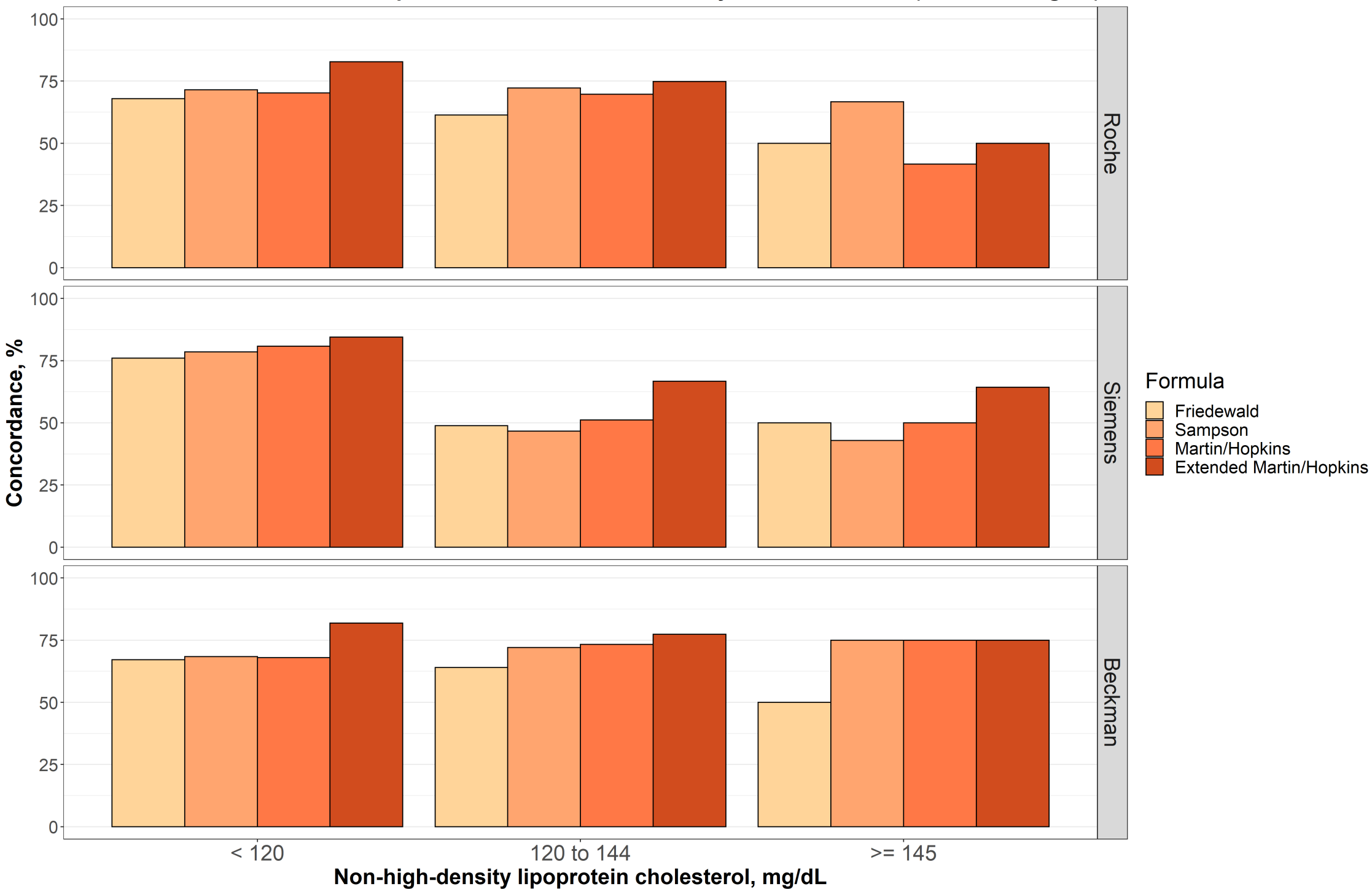

Supplement: Supplemental Information 6 [file peerj-11-14544-s006.pdf]

Concordances of the different equations for LDL-C estimation by nonHDL-C strata (LDL  $\geq$  130 mg/dL)

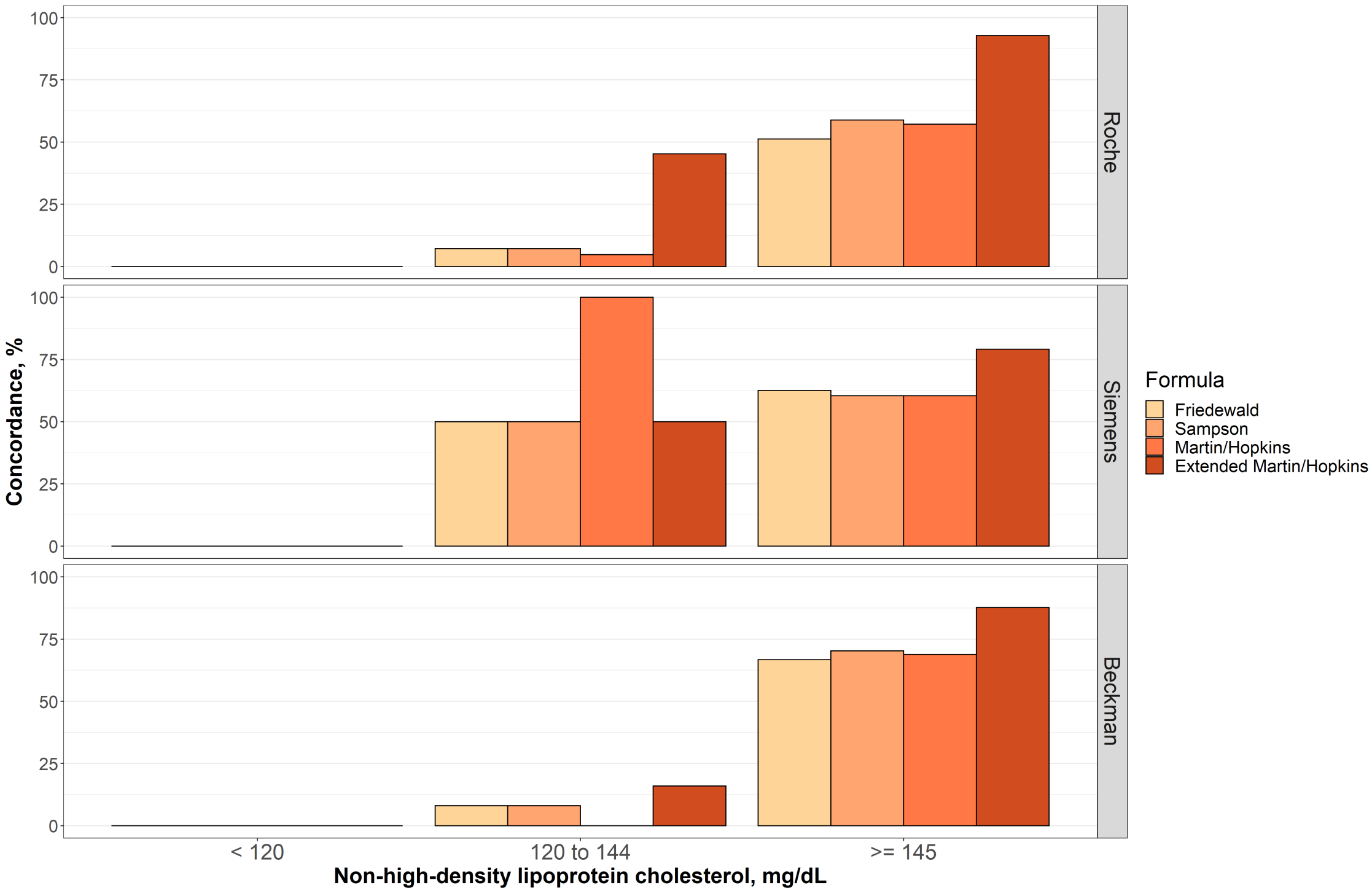

Supplement: Supplemental Information 8 [file peerj-11-14544-s008.pdf]

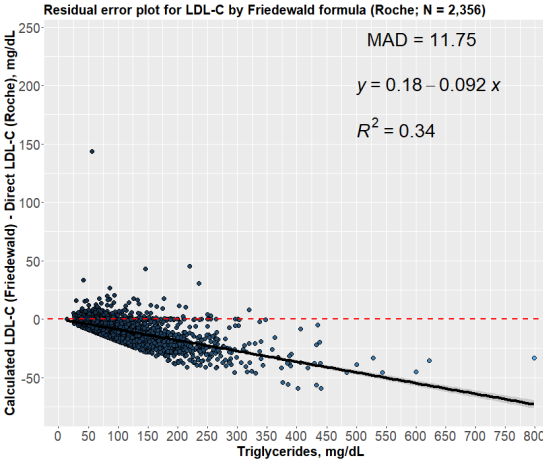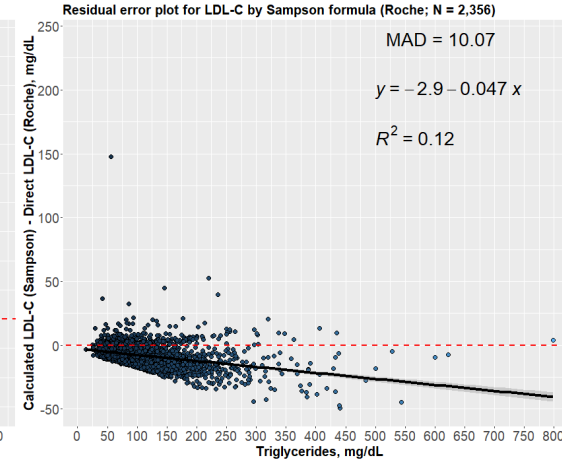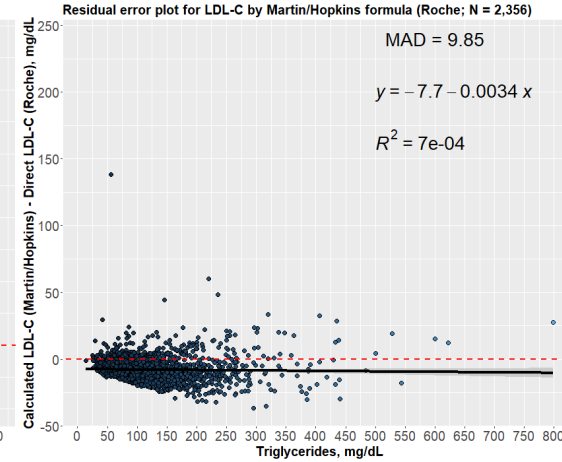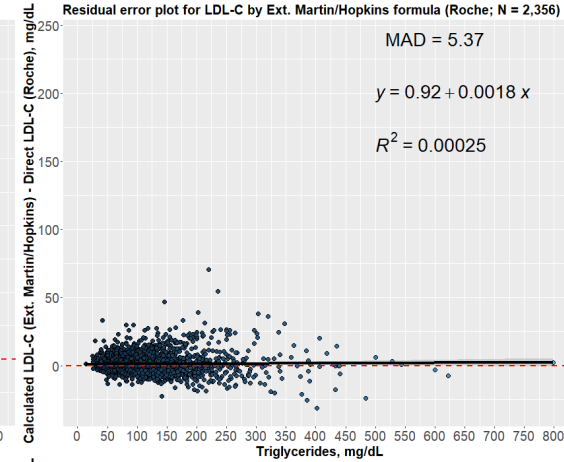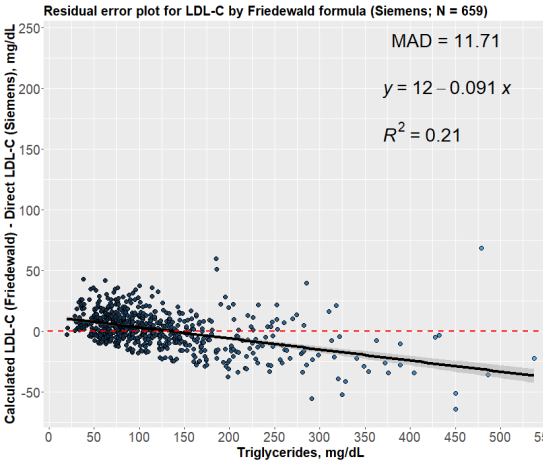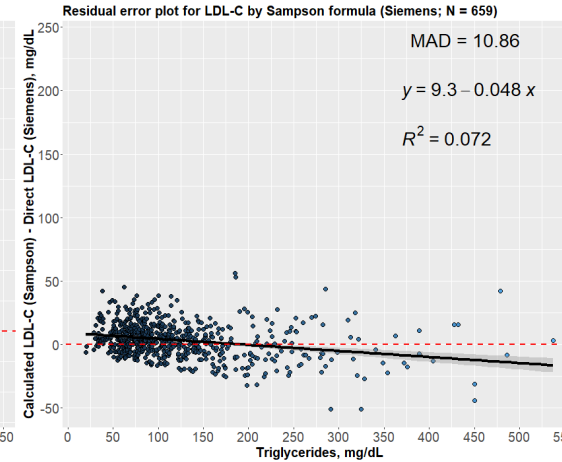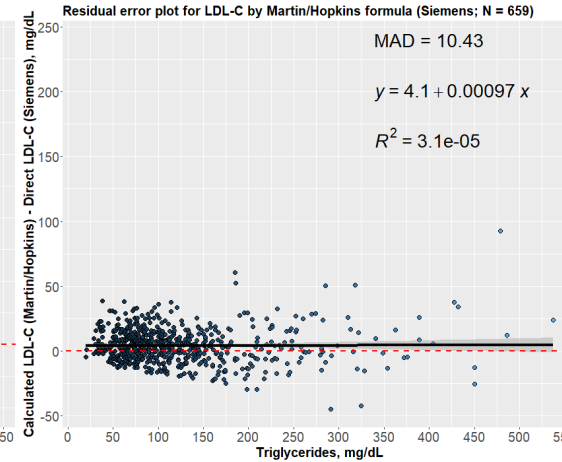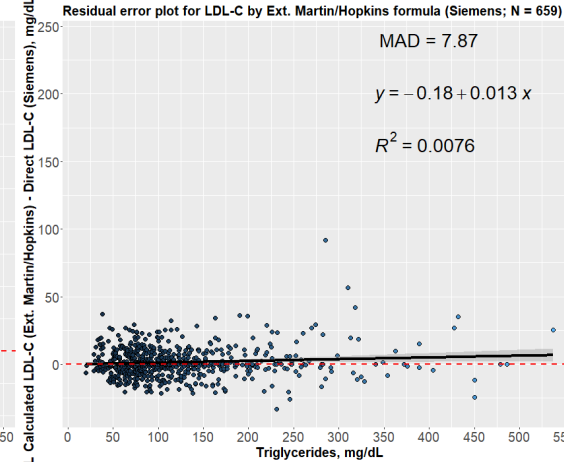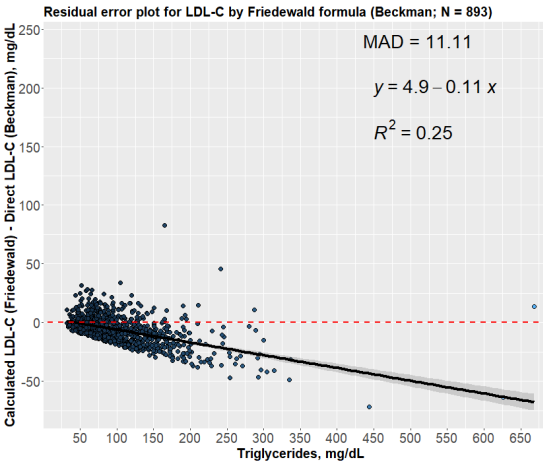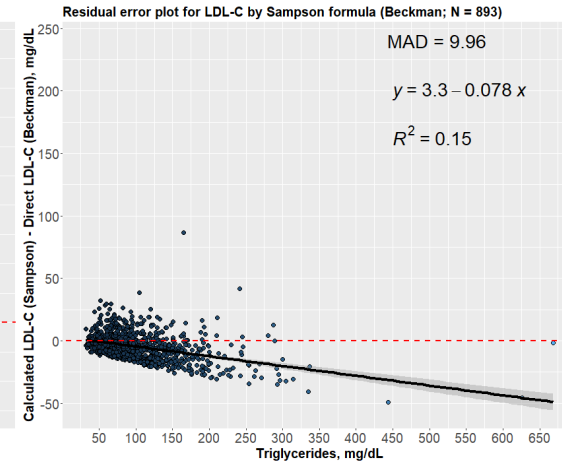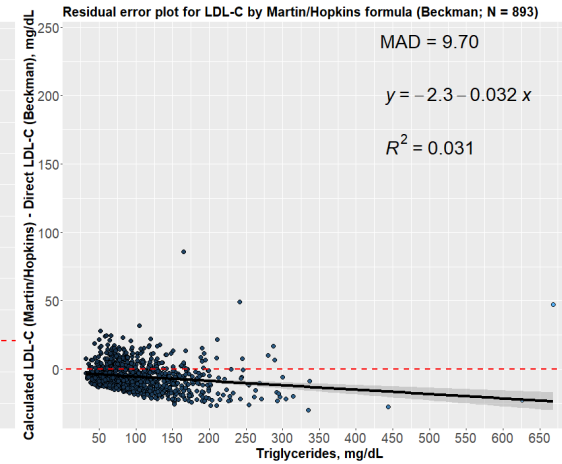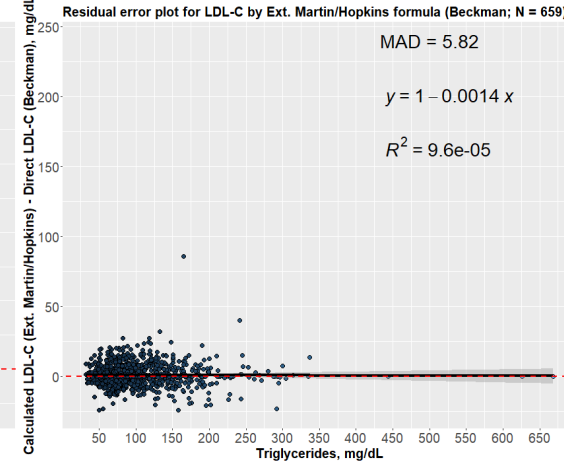

Supplement: Supplemental Information 9 [file peerj-11-14544-s009.pdf]

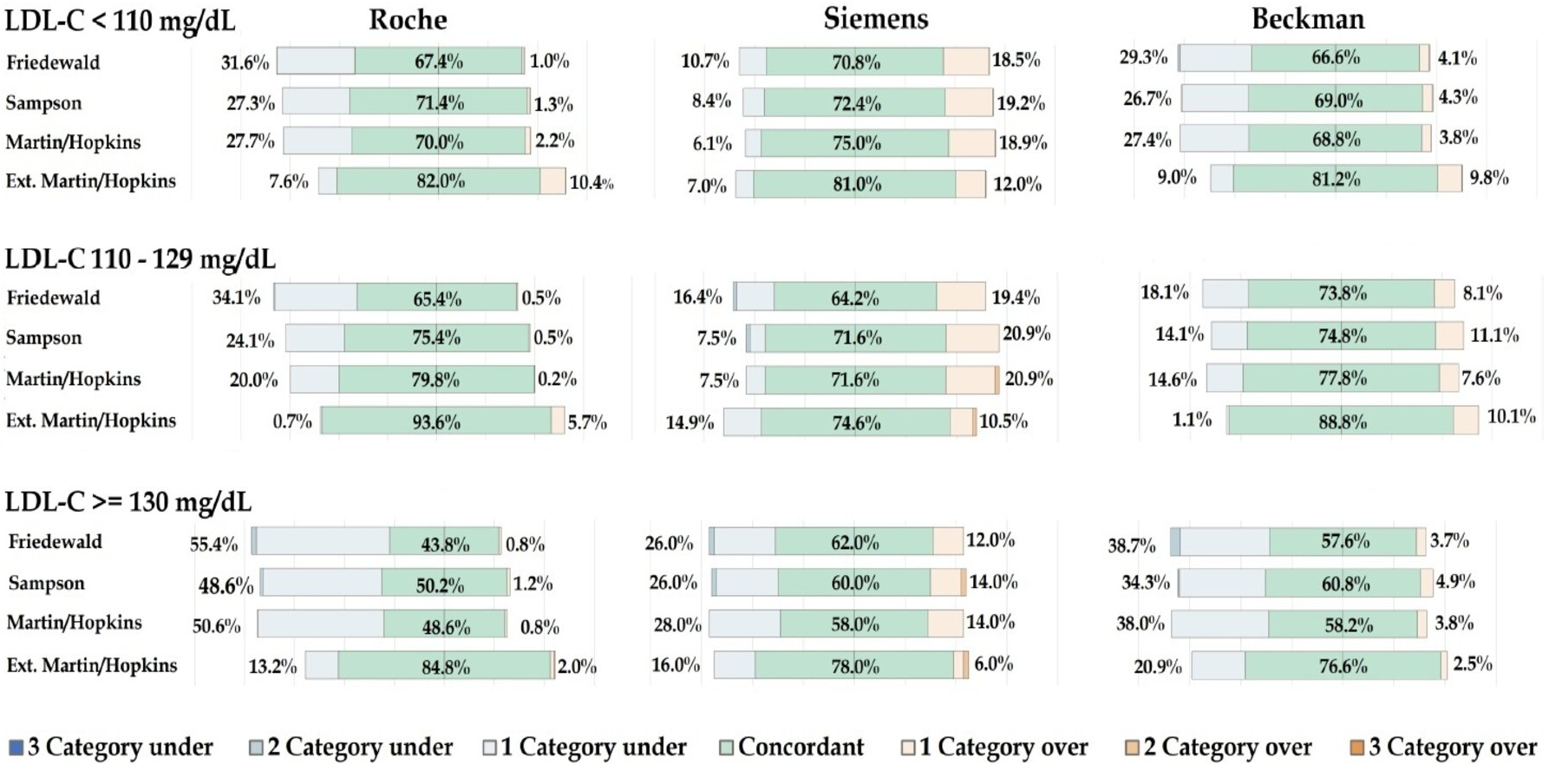

Supplement: Supplemental Information 10 [file peerj-11-14544-s010.png]
