# Supplementary material for: Validation of low-density lipoprotein cholesterol equations in pediatric population
Source: PeerJ. 2023 Jan 5;11:e14544. doi: 10.7717/peerj.14544 (PMC9826611; doi:10.7717/peerj.14544)

Concordances of the different equations for LDL-C estimation by triglycerides strata (LDL < 110 mg/dL)

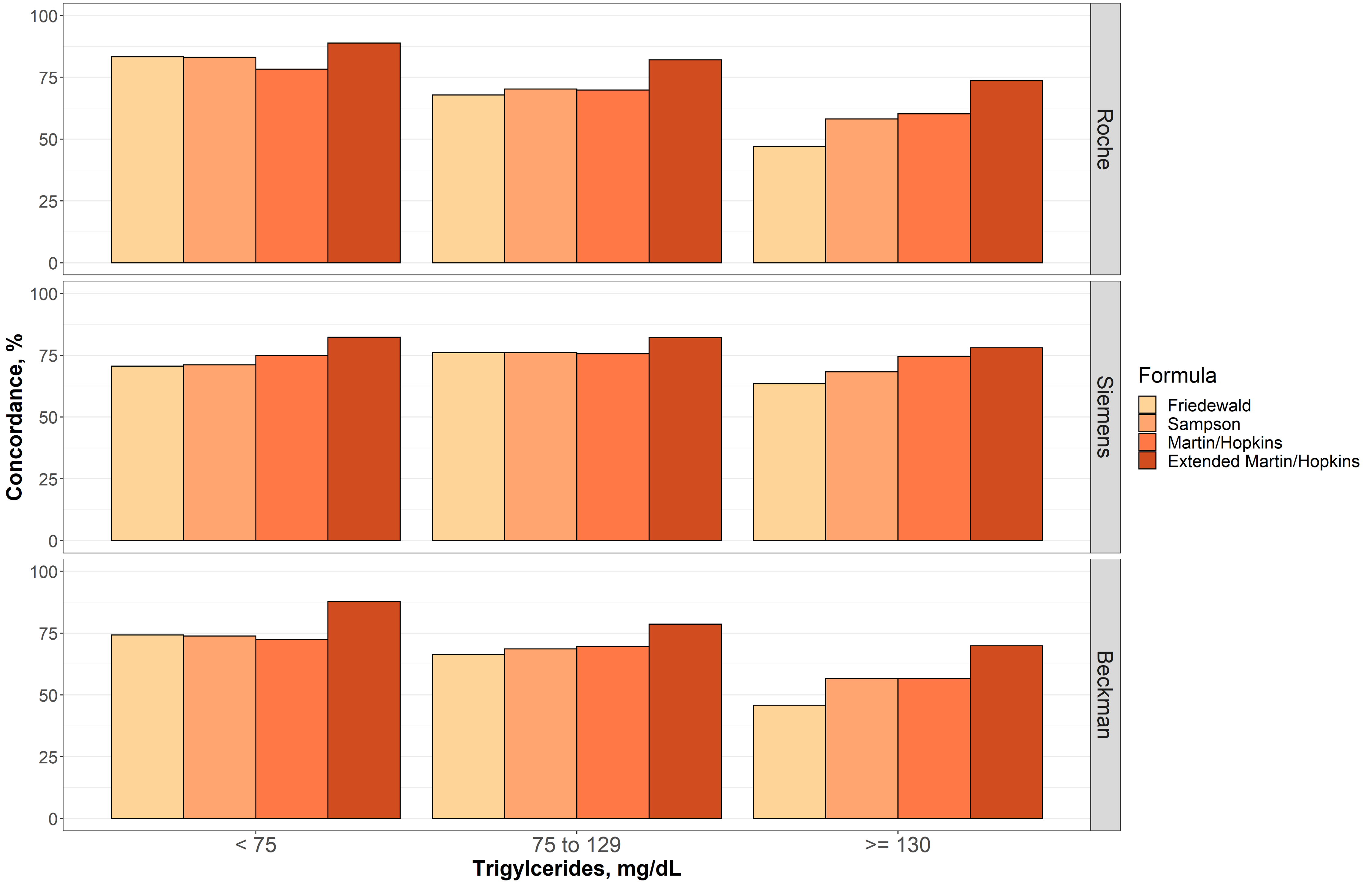

Supplement: Supplemental Information 2 [file peerj-11-14544-s002.pdf]
