# Supplementary material for: Validation of low-density lipoprotein cholesterol equations in pediatric population
Source: PeerJ. 2023 Jan 5;11:e14544. doi: 10.7717/peerj.14544 (PMC9826611; doi:10.7717/peerj.14544)
Supplement: Supplemental Information 4 [file peerj-11-14544-s004.pdf]

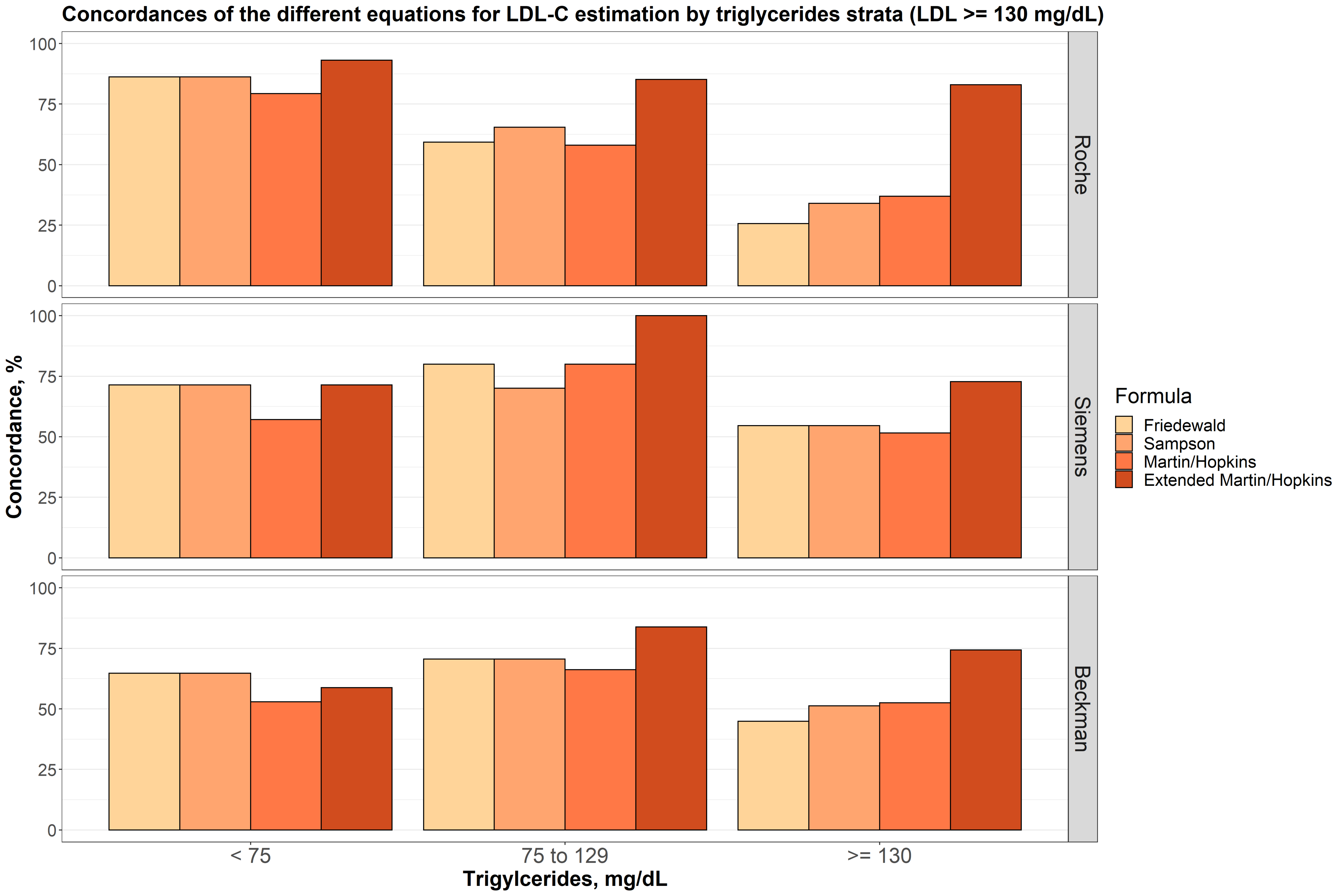

Concordances of the different equations for LDL-C estimation by triglycerides strata (LDL >= 130 mg/dL)
Concordance, %
Trigylcerides, mg/dL
< 75
75 to 129
>= 130
Roche
Siemens
Beckman
0
25
50
75
100
Formula
Friedewald
Sampson
Martin/Hopkins
Extended Martin/Hopkins
