# Supplementary material for: Validation of low-density lipoprotein cholesterol equations in pediatric population
Source: PeerJ. 2023 Jan 5;11:e14544. doi: 10.7717/peerj.14544 (PMC9826611; doi:10.7717/peerj.14544)
Supplement: Supplemental Information 11 — Median statistics for the ratio of triglycerides to very low-density lipoprotein cholesterol by the cross table of non-high-density lipoprotein cholesterol and triglycerides calculated from the Turkish population (calculated for each direct assay method for 180-cell strata) [file peerj-11-14544-s011.docx]

**Table 2. Median statistics for the ratio of triglycerides to very low-density lipoprotein cholesterol by the cross table of non-high-density lipoprotein cholesterol and triglycerides calculated from the Turkish population (calculated for each direct assay method for 180-cell strata)**

| **TG Levels  (mg/dL)** | **Non-HDL-C (mg/dL)** | | | | | |
| --- | --- | --- | --- | --- | --- | --- |
|  | **< 100** | **100 - 129** | **130 - 159** | **160 - 189** | **190 - 219** | **≥ 220** |
| **7-49** | **R:**7.88 **S:**2.65 **B:**7.00 | **R:**8.20 **S:**2.52 **B:**3.80 | **R:**4.16 **S:**2.87 **B:**2.61 | **R:**4.90 **S:-** **B:**2.35 | **R:-** **S:-** **B:-** | **R:-** **S:-** **B:-** |
| **50-56** | **R:**9.17 **S:**3.33 **B:**10.20 | **R:**5.80 **S:**2.53 **B:**5.21 | **R:**3.47 **S:**1.40 **B:**1.71 | **R:-** **S:**3.89 **B:-** | **R:-** **S:**4.58 **B:-** | **R:**0.36 **S:-** **B:-** |
| **57-61** | **R:**10.00 **S:**3.45 **B:**10.90 | **R:**5.55 **S:**5.78 **B:**6.56 | **R:**13.06 **S:-** **B:**3.84 | **R:**5.10 **S:-** **B:**1.49 | **R:-** **S:**6.95 **B:-** | **R:**14.25 **S:-** **B:-** |
| **62-66** | **R:**10.58 **S:**3.94 **B:**8.55 | **R:**10.50 **S:**2.80 **B:**4.57 | **R:**5.00 **S:**2.29 **B:**3.90 | **R:**5.33 **S:-** **B:**2.32 | **R:-** **S:-** **B:-** | **R:**5.91 **S:**1.15 **B:-** |
| **67-71** | **R:**11.83 **S:**3.50 **B:**11.67 | **R:**8.75 **S:**3.66 **B:**5.05 | **R:**10.58 **S:**5.85 **B:**2.35 | **R:**3.39 **S:**1.74 **B:-** | **R:-** **S:-** **B:-** | **R:-** **S:-** **B:-** |
| **72-75** | **R:**12.17 **S:**3.56 **B:**12.33 | **R:**9.00 **S:**3.27 **B:**4.80 | **R:**6.64 **S:**3.60 **B:**3.60 | **R:**2.57 **S:-** **B:**10.48 | **R:-** **S:-** **B:-** | **R:-** **S:-** **B:-** |
| **76-79** | **R:**8.67 **S:**5.55 **B:**14.69 | **R:**9.82 **S:**2.73 **B:**6.24 | **R:**6.50 **S:**1.92 **B:**5.92 | **R:-** **S:-** **B:-** | **R:-** **S:**1.49 **B:**8.44 | **R:-** **S:-** **B:-** |
| **80-83** | **R:**13.50 **S:**4.03 **B:**11.96 | **R:**6.75 **S:**3.77 **B:**7.43 | **R:**5.00 **S:**3.93 **B:**5.97 | **R:-** **S:-** **B:**5.33 | **R:**4.88 **S:-** **B:-** | **R:-** **S:-** **B:-** |
| **84-87** | **R:**10.50 **S:**3.57 **B:**4.78 | **R:**12.00 **S:**4.43 **B:**8.55 | **R:**5.15 **S:**3.47 **B:**4.89 | **R:**9.33 **S:-** **B:-** | **R:-** **S:-** **B:**5.73 | **R:**1.95 **S:-** **B:-** |
| **88-92** | **R:**10.00 **S:**4.29 **B:**15.25 | **R:**10.50 **S:**4.09 **B:**9.10 | **R:**6.92 **S:**3.29 **B:**13.35 | **R:**4.94 **S:**1.86 **B:**3.35 | **R:**5.10 **S:**5.68 **B:-** | **R:-** **S:-** **B:**3.42 |
| **93-96** | **R:**13.71 **S:**7.27 **B:**10.44 | **R:**11.69 **S:**4.36 **B:**5.99 | **R:**10.44 **S:**2.10 **B:**4.43 | **R:**3.80 **S:**1.85 **B:**4.60 | **R:-** **S:-** **B:**4.41 | **R:**2.82 **S:-** **B:-** |
| **97-100** | **R:**12.38 **S:**7.54 **B:**19.60 | **R:**11.11 **S:**4.24 **B:**9.90 | **R:**11.11 **S:**2.49 **B:**6.19 | **R:**11.11 **S:**3.63 **B:**6.47 | **R:**6.53 **S:-** **B:-** | **R:-** **S:-** **B:-** |
| **101-105** | **R:**11.67 **S:**5.00 **B:**13.00 | **R:**7.77 **S:**4.04 **B:**6.93 | **R:**11.44 **S:**3.23 **B:**10.20 | **R:**3.78 **S:**3.22 **B:**3.29 | **R:**7.43 **S:-** **B:**13.13 | **R:-** **S:-** **B:**1.91 |
| **106-110** | **R:**10.65 **S:**6.11 **B:**15.43 | **R:**11.78 **S:**4.68 **B:**10.80 | **R:**8.01 **S:**12.02 **B:**4.23 | **R:**10.90 **S:**22.00 **B:-** | **R:-** **S:-** **B:**2.82 | **R:**13.20 **S:-** **B:-** |
| **111-115** | **R:**11.40 **S:**5.33 **B:**56.75 | **R:**13.88 **S:**4.31 **B:**10.96 | **R:**9.33 **S:**4.28 **B:**8.08 | **R:**21.83 **S:**3.85 **B:-** | **R:-** **S:-** **B:**4.87 | **R:**5.80 **S:-** **B:**8.54 |
| **116-120** | **R:**9.54 **S:**5.67 **B:**34.42 | **R:**10.91 **S:**3.66 **B:**13.28 | **R:**13.81 **S:**4.13 **B:**9.23 | **R:**5.95 **S:**2.98 **B:**7.50 | **R:**8.43 **S:-** **B:**5.14 | **R:-** **S:-** **B:-** |
| **121-126** | **R:**13.94 **S:**7.59 **B:**12.10 | **R:**12.45 **S:**6.65 **B:**12.01 | **R:**10.42 **S:**3.97 **B:**5.39 | **R:**5.04 **S:**2.59 **B:-** | **R:**4.10 **S:-** **B:**5.68 | **R:-** **S:-** **B:-** |
| **127-132** | **R:**11.32 **S:**10.92 **B:**15.88 | **R:**11.00 **S:**5.70 **B:**14.56 | **R:**10.92 **S:**4.21 **B:**7.47 | **R:**7.23 **S:**7.81 **B:**5.27 | **R:-** **S:-** **B:**4.90 | **R:-** **S:-** **B:-** |
| **133-138** | **R:**9.39 **S:**7.82 **B:**22.18 | **R:**8.56 **S:**5.45 **B:**14.78 | **R:**13.45 **S:**3.68 **B:**11.33 | **R:**10.42 **S:**4.44 **B:**4.39 | **R:-** **S:-** **B:**4.22 | **R:-** **S:-** **B:-** |
| **139-146** | **R:**13.69 **S:**6.86 **B:**14.20 | **R:**12.03 **S:**5.31 **B:**13.74 | **R:**10.69 **S:**4.44 **B:**6.66 | **R:**5.80 **S:**6.38 **B:**6.57 | **R:-** **S:-** **B:-** | **R:-** **S:**6.32 **B:-** |
| **147-154** | **R:**12.54 **S:**5.17 **B:**12.58 | **R:**12.58 **S:**4.90 **B:**14.88 | **R:**10.46 **S:**7.78 **B:**8.22 | **R:**9.80 **S:**3.36 **B:**10.20 | **R:-** **S:-** **B:**5.16 | **R:-** **S:-** **B:**7.19 |
| **155-163** | **R:**11.98 **S:**5.85 **B:**7.90 | **R:**10.51 **S:**6.35 **B:**15.42 | **R:**9.65 **S:**4.85 **B:**9.24 | **R:**9.38 **S:**13.19 **B:**5.03 | **R:-** **S:-** **B:-** | **R:**9.53 **S:**14.46 **B:-** |
| **164-173** | **R:**12.29 **S:**8.22 **B: -** | **R:**10.17 **S:**7.31 **B:**9.61 | **R:**13.49 **S:**10.12 **B:**15.64 | **R:**8.68 **S:**4.26 **B:**5.45 | **R:**6.30 **S:**6.81 **B:**6.00 | **R:**6.96 **S:-** **B:-** |
| **174-185** | **R:**13.16 **S:**8.81 **B:**18.43 | **R:**12.50 **S:**5.62 **B:**12.74 | **R:**9.05 **S:**5.58 **B:**7.38 | **R:**13.69 **S:-** **B:-** | **R:**4.94 **S:**23.65 **B:-** | **R:**3.98 **S:**1.91 **B:-** |
| **186-201** | **R:**12.93 **S:**14.49 **B:-** | **R:**10.53 **S:**8.50 **B:**15.92 | **R:**11.38 **S:**52.47 **B:**7.17 | **R:**9.21 **S:**7.86 **B:**5.76 | **R:**6.33 **S:-** **B:**4.64 | **R:-** **S:**2.51 **B:**3.65 |
| **202-220** | **R:**12.06 **S:**13.45 **B:**21.9 | **R:**9.90 **S:**7.74 **B:**23.78 | **R:**10.35 **S:**6.60 **B:**7.89 | **R:**10.68 **S:**6.95 **B:**3.70 | **R:**15.62 **S:**26.13 **B:-** | **R:**5.37 **S:**3.24 **B:-** |
| **221-247** | **R:**10.06 **S:**8.16 **B:**17.15 | **R:**11.45 **S:**6.74 **B:**22.81 | **R:**11.19 **S:**5.58 **B:**8.19 | **R:**8.00 **S:**4.33 **B:**11.60 | **R:**20.07 **S:**7.00 **B:**5.90 | **R:**5.75 **S:-** **B:**4.48 |
| **248-292** | **R:**7.93 **S:**9.37 **B:**63.50 | **R:**9.11 **S:**7.21 **B:**11.13 | **R:**10.12 **S:**7.05 **B:**13.65 | **R:**10.80 **S:**5.40 **B:**5.67 | **R:**7.37 **S:**53.43 **B:-** | **R:**6.33 **S:**3.85 **B:**4.22 |
| **293-399** | **R:**8.78 **S:**6.63 **B:-** | **R:**8.97 **S:**8.86 **B:**24.42 | **R:**8.30 **S:**6.59 **B:**14.93 | **R:**9.10 **S:**7.72 **B:**18.61 | **R:**7.88 **S:-** **B:-** | **R:**13.70 **S:**14.48 **B:**7.93 |
| **≥400** | **R:**8.84 **S:-** **B:-** | **R:**7.67 **S:**7.97 **B:**26.12 | **R:**9.09 **S:**8.99 **B:-** | **R:**6.44 **S:-** **B:-** | **R:**12.58 **S:-** **B:**10.26 | **R:**8.71 **S:**2.92 **B:**4.54 |

**TG: triglycerides; HDL-C: high-density lipoprotein cholesterol; R: Roche; B: Beckman; S: Siemens. Median statistic were not calculated since there were no samples in this case.**
